# A traumatic arteriovenous fistula causing venous insufficiency

**DOI:** 10.1093/jscr/rjae535

**Published:** 2024-08-29

**Authors:** John Shelton, Amanthana Marasinghe

**Affiliations:** Professorial Surgical Unit, Teaching Hospital Jaffna, Jaffna, 40000, Srilanka; Vascular and Transplant Unit, Colombo North Teaching Hospital, Ragama, 11010, Srilanka

**Keywords:** chronic venous ulcers, chronic venous hypertension, arteriovenous fistula, venogram, endovascular techniques

## Abstract

Chronic leg ulcers are one of the clinical manifestations of untreated traumatic arteriovenous fistula (AVF). Ulcers due to secondary venous etiology like AVF are rare and easily missed. We present the case of a 31-year-old male who sustained a gunshot injury to the knee 12 years ago. Despite the absence of varicose veins, he presented with a neglected venous ulcer persisting for 2 years. A computed tomographic venogram revealed an AVF in the supra-genicular popliteal artery and vein. The patient subsequently underwent open surgical correction. Complete ulcer healing was achieved in 6 months. This case underscores the potential for a high-flow post-traumatic AVF to induce a non-healing venous ulcer even in the absence of apparent varicose veins. Secondary causes of chronic venous diseases are often overlooked, leading to complications. Early recognition is crucial to preventing further morbidity.

## Introduction

Nonhealing chronic leg ulcers, defined as a persistent breach in the epithelial integrity of the skin between the malleoli and knee lasting >6 weeks, are frequently encountered in clinical practice. The etiology of these ulcers is often multifactorial, with vascular issues playing a predominant role. Chronic venous hypertension, commonly arising from incompetence in superficial and/or deep veins due to faulty valves, is a primary cause of enduring leg ulcers [1].

Venous obstruction resulting from an arteriovenous fistula (AVF) stands out as a notable secondary cause of chronic venous disease. During the initial stages, venous hypertension may remain asymptomatic, but prolonged inflammation can lead to skin thickening, scaling, and eventual ulceration.

This case report presents a patient with a persistent venous ulcer associated with secondary chronic venous disease, managed for several years without addressing the underlying etiology. Ultimately, the patient was diagnosed with a traumatic AVF, underscoring the importance of identifying and treating secondary causes in cases of chronic venous ulcers.

## Case presentation

**Figure 1:**
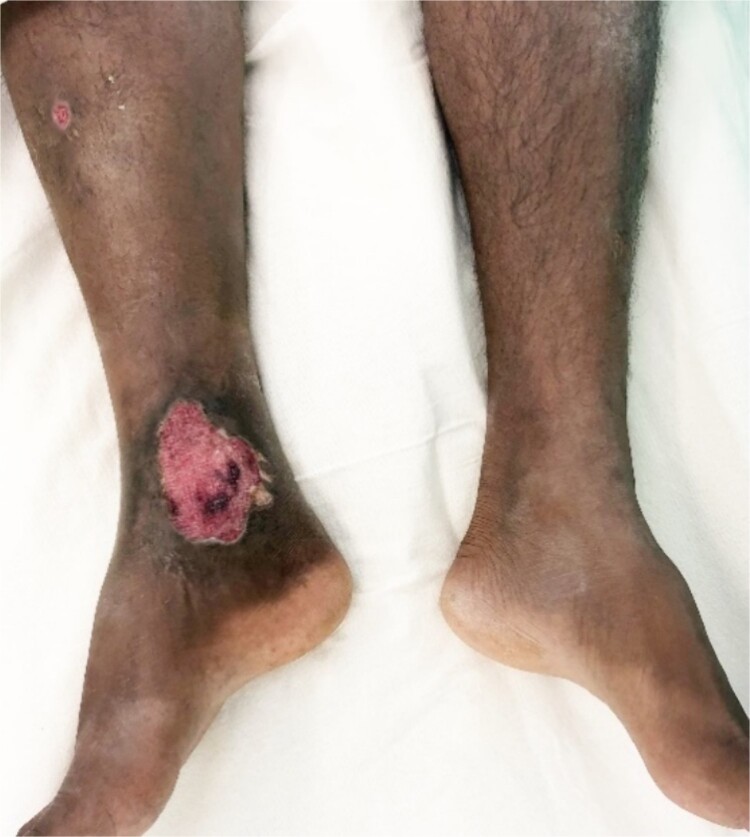
A neglected venous ulcer in the right gaiter area for 2 years.

A 31-year-old military officer sought consultation at the vascular and transplant unit. Presenting with a chronic non-healing ulcer on the right lower limb gaiter area along with noticeable skin pigmentation (Fig. 1), persisting for a duration of 2 years. The patient’s medical history revealed a gunshot injury to the right knee region in 2008, without reported fractures or major vascular injuries at the time of the incident. Initial treatment took place at a military hospital, involving no significant surgical interventions.

The patient was evaluated for chronic leg ulcer under the care of a surgical unit. Bilateral lower limb venous duplex scans conducted during the earlier evaluation showed no features of varicose veins or chronic venous insufficiency in the superficial lower limb veins. Notably, an ulcer edge biopsy at that time was consistent with stasis dermatitis, leading to the initiation of a management plan involving four-layer strapping. The patient, devoid of significant comorbidities and without any family history of similar ailments, continued this management protocol.

**Figure 2:**
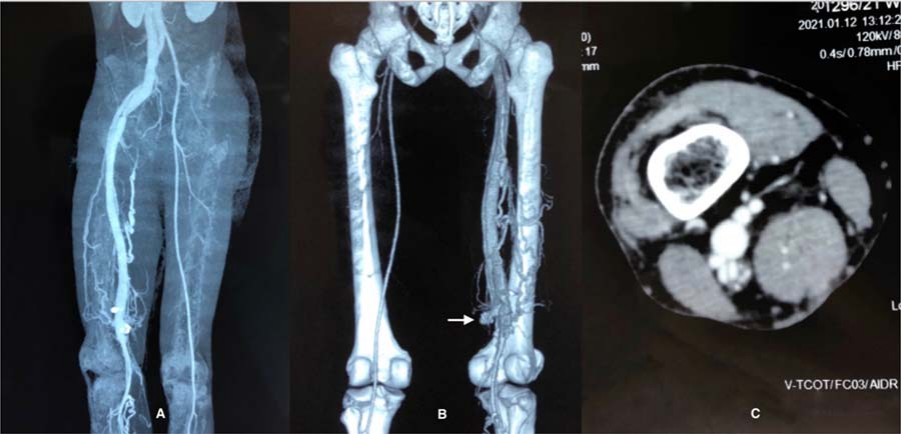
CT venogram (A) axial, (B) 3D reconstruction, (C) axial view at adductor cannel. There is early opacification of the popliteal vein in the arterial phase just below the level of adductor cannel on the right side and it is draining into inferior vena cava via the common femoral vein and iliac vein. The superficial venous system is not dilated. These features are consistent with high flow AVF most probably between the popliteal artery and vein.

On our examination, the superficial veins did not reveal obvious varicosities, and there were no signs indicative of heart failure. Distal pedal pulses were palpable. Routine inflammatory markers were within normal ranges, and a bilateral lower limb venous duplex scan showed no abnormalities. However, a color Doppler examination revealed the presence of an AVF in the supra-genicular region. Further clarification of the lesion was sought through a computed contrast-enhanced tomographic venogram (Fig. 2), which conclusively identified a high-flow AVF located between the popliteal artery and vein.

**Figure 3:**
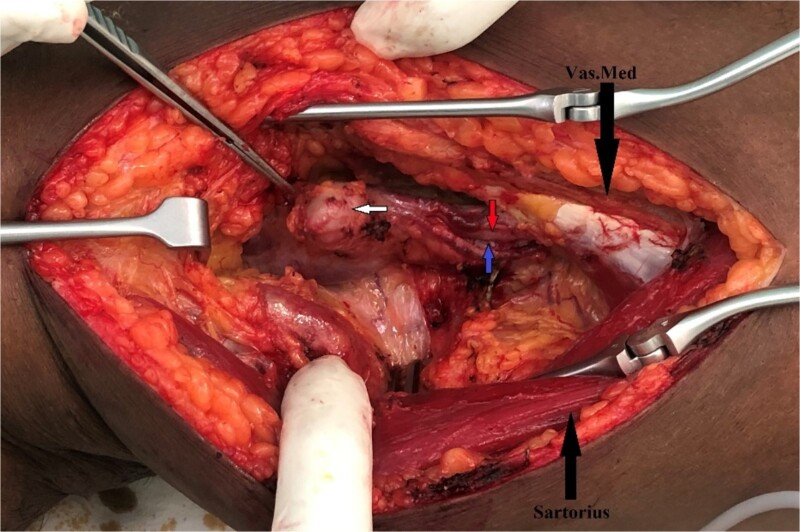
Intraoperative finding of AVF with aneurysm between popliteal artery and vein.

Open surgical correction of AVF was performed by medial supra geniculate approach, and exploration of neurovascular structures and the AVF was undertaken (Fig. 3). The popliteal artery disconnected from the supra-genicular popliteal artery from the AVF was executed and end-to-end repair of the artery was subsequently performed. The patient exhibited an uneventful recovery.

During the initial follow-up, conducted 4 weeks postoperatively, duplex scanning unequivocally affirmed the successful closure of the AVF. Subsequent monitoring at the 3-month mark revealed a notable reduction in ulcer size, accompanied by the presence of healthy granulation tissue. Notably, no limb edema was observed during this follow-up period. This positive progression in the patient’s condition underscored the efficacy of the surgical intervention and the favorable postoperative course.

## Discussion and conclusion

AVFs are abnormal permanent connections between arteries and veins, bypassing the normal capillary flow. They can result from congenital or traumatic origins, with the latter further classified into iatrogenic and traumatic categories. Iatrogenic fistulas often arise from invasive medical procedures such as catheter insertions, surgeries, and biopsies, while accidental fistulas are typically associated with penetrating wounds, including stab or gunshot injuries. Notably, posttraumatic AVFs predominantly involve the lower extremities, and spontaneous resolution is a rare occurrence, observed in only ~2% of cases [2].

Clinical manifestations of posttraumatic AVFs usually manifest shortly after the traumatic incident, but delayed presentations, as seen in this case, have been documented [3]. Recognizable signs include a thrill and a murmur due to turbulent blood flow, along with peripheral indications like a weak distal pulse, edema, cyanosis, and pallor. Advanced cases may exhibit features of high-output heart failure, and skin ulceration can occur due to ischemia from blood diversion through the fistula or venous hypertension. In this instance, the mechanism likely involves chronic venous insufficiency given the patient’s existing chronic venous skin changes (C6EsAdPo).

Diagnosing chronic venous disease secondary to AVF can be challenging without a detailed history of penetrating trauma. Suspecting AV fistula is crucial in cases where ulcers resist standard treatment. Color Doppler ultrasound serves as the initial assessment tool for traumatic AVFs, with computed tomography (CT) angiography confirming findings and detailing the exact fistula site and hemodynamics. Digital subtraction angiography remains the gold standard for diagnosis and management, offering comprehensive information on location, type of fistula, affected vessels, and perivascular complications [3, 4].

In terms of management, the options for traumatic AVFs include surgery or endovascular techniques. In hemodynamically stable patients, minimally invasive endovascular techniques have become the gold standard due to advantages such as reduced pain, quicker recovery, and a lower infection risk from remote access sites. Treatment involves embolization with various agents, including autologous blood clots, gel foam sponges, microfibrillar collagen, polyvinyl alcohol sponges, coils, detachable and nondetachable balloons, and cyanoacrylate [5].

In the contemporary medical landscape, minimally invasive endovascular techniques have surpassed open surgical correction as the preferred approach for hemodynamically stable patients with traumatic AVFs. These techniques offer notable benefits, including reduced morbidity, faster recovery, and decreased infection risk. However, due to resource limitations and the absence of an endovascular suite, open surgical correction remains a viable option for hemodynamically unstable patients.

This case highlights the importance of considering traumatic AVFs as potential contributors to non-healing venous ulcers, even in the absence of overt varicose veins. The often missed secondary causes of chronic venous diseases can lead to complications necessitating timely intervention. Recognition of these complexities and the appropriate selection of diagnostic and therapeutic modalities are crucial for achieving favorable patient outcomes.

## Data Availability

The datasets used and/or analyzed during the current study are available from the corresponding author upon reasonable request. All data generated or analyzed during this study are included in this article.
